# Estimating the Impact of Expanding Treatment Coverage and Allocation Strategies for Chronic Hepatitis C in a Direct Antiviral Agent Era

**DOI:** 10.1371/journal.pone.0163095

**Published:** 2016-09-15

**Authors:** Kittiyod Poovorawan, Wirichada Pan-ngum, Lisa J. White, Ngamphol Soonthornworasiri, Polrat Wilairatana, Rujipat Wasitthankasem, Pisit Tangkijvanich, Yong Poovorawan

**Affiliations:** 1 Department of Clinical Tropical Medicine, Faculty of Tropical Medicine, Mahidol University, Bangkok, Thailand; 2 Department of Tropical Hygiene, Faculty of Tropical Medicine, Mahidol University, Bangkok, Thailand; 3 Mathematical and Economic Modelling Group, Mahidol-Oxford Tropical Medicine Research Unit, Faculty of Tropical Medicine, Mahidol University, Bangkok, Thailand; 4 Centre for Tropical Medicine, Nuffield Department of Medicine, University of Oxford, Oxford, UK; 5 Department of Pediatrics, Center of Excellence in Clinical Virology, Faculty of Medicine, Chulalongkorn University, Bangkok, Thailand; 6 Department of Biochemistry, Research Unit of Hepatitis and Liver Cancer, Faculty of Medicine, Chulalongkorn University, Bangkok, Thailand; University of New South Wales, AUSTRALIA

## Abstract

Hepatitis C virus (HCV) infection is an important worldwide public health problem, and most of the global HCV burden is in low- to middle-income countries. This study aimed to estimate the future burden of chronic hepatitis C (CHC) and the impact of public health policies using novel antiviral agents in Thailand. A mathematical model of CHC transmission dynamics was constructed to examine the disease burden over the next 20 years using different treatment strategies. We compared and evaluated the current treatment (PEGylated interferon and ribavirin) with new treatments using novel direct-acting antiviral agents among various treatment policies. Thailand’s CHC prevalence was estimated to decrease 1.09%–0.19% in 2015–2035. Expanding treatment coverage (i.e., a five-fold increment in treatment accessibility) was estimated to decrease cumulative deaths (33,007 deaths avoided, 25.5% reduction) from CHC-related decompensated cirrhosis and hepatocellular carcinoma (HCC). The yearly incidence of HCC-associated HCV was estimated to decrease from 2,305 to 1,877 cases yearly with expanding treatment coverage. A generalized treatment scenario (i.e., an equal proportional distribution of available treatment to individuals at all disease stages according to the number of cases at each stage) was predicted to further reduce death from HCC (9,170 deaths avoided, 11.3% reduction) and the annual incidence of HCC (i.e., a further decrease from 1,877 to 1,168 cases yearly, 37.7% reduction), but cumulative deaths were predicted to increase (by 3,626 deaths, 3.7% increase). Based on the extensive coverage scenario and the generalized treatment scenario, we estimated near-zero death from decompensated cirrhosis in 2031. In conclusion, CHC-related morbidity and mortality in Thailand are estimated to decrease dramatically over the next 20 years. Treatment coverage and allocation strategies are important factors that affect the future burden of CHC in resource-limited countries like Thailand.

## Introduction

Hepatitis C virus (HCV) infection is an important worldwide public health problem [1]. Most HCV cases develop into chronic hepatitis C (CHC), which may progress to liver fibrosis, cirrhosis, hepatocellular carcinoma, and death [2]. Researchers have calculated that 130–170 million people are infected with HCV (global prevalence: 2%–3%) [3]. Approximately 75%–85%, 60%–70%, and 5%–20% of people infected with HCV will develop chronic hepatitis, hepatic steatosis/fibrosis, and cirrhosis, respectively. Additionally, 1%–5% of people acutely infected with HCV will progress to life-threatening complications and hepatocellular carcinoma (HCC) within 20 years [4]. Recent total global viremic HCV infections have been estimated at 80 (64–103) million, with the predominant HCV genotypes being 1 (46%) and 3 (22%) [5]. More than 80% of the global HCV burden is in low- to middle-income countries [6].

Despite great successes in virology and diagnostics, several difficulties have prevented improvement in HCV infection control and elimination in the new era. New HCV infections still occur, especially in poor regions, where HCV can be endemic, and long-term sequelae cause growing economic and health burdens [7]. Recently, there have been major scientific advances for HCV treatment, such as new direct-acting antivirals (DAAs), which have higher cure rates and fewer side effects than polyethylene glycolated (PEGylated) interferon and ribavirin. Some of them are pan-genotypic drugs, but they are only available at considerably high costs [8]. These advances have led to the potential of HCV treatment delivery to national public health programs and decreased overall HCV-related morbidity and mortality [9]. This new treatment is also cost-effective in some developed countries [10, 11]. New DAAs that can cure HCV infection have been approved in countries like the United States [8]. However, these new agents are still limited and unavailable in many countries, including Thailand.

In Thailand, 2.2% of individuals from four geographically distinct provinces had positive anti-HCV in 2004 [12]. Seroprevalence was 0.51%–0.98% among first-time blood donors who were screened by the National Blood Center but 70%–90% in high-risk groups, such as people who inject drugs [13, 14]. Projection data on CHC’s burden and the effects of public health policies are beneficial, especially in the new treatment era, as they can help to develop public health programs to control and eliminate HCV infection in Thailand and throughout Southeast Asia. The objective of this study was to estimate the future burden of CHC and public health benefits of CHC treatment using different treatment strategies in Thailand.

## Materials and Methods

**Fig 1:**
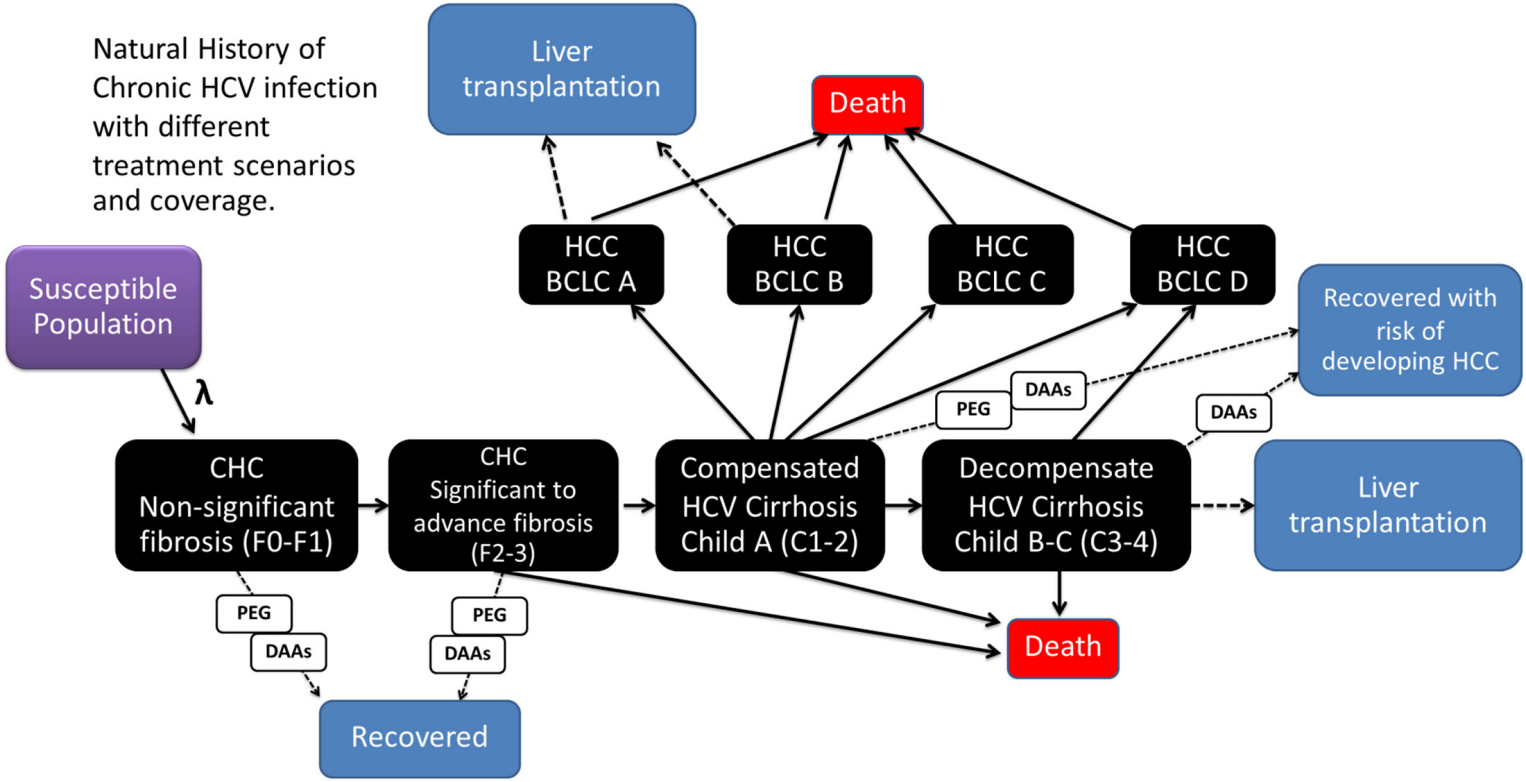
Study design of the transmission and disease progression model. The fibrosis stage of progression develops gradually, and cirrhosis and decompensation develop over time. The risk of developing HCC starts after cirrhosis. Survival rates depend on the severity of disease at each stage. The current standard treatment in Thailand was compared with new direct-acting antivirals with different treatment coverage and allocations. *HCV-related mortality.

We analyzed HCV data from national epidemiological surveys and available published literature on HCV, including prevalence, susceptible populations, transmission, genotypic distribution, degree of liver fibrosis, progression of CHC, efficacy of treatments, cost of treatments, and treatment coverage (Table 1). The target population comprised patients infected with HCV from Thailand. Their epidemiological data were evaluated over a lifetime horizon using the Markov model (Fig 1). All cases entered into the model had the possibility of death from general, non-HCV-related causes (Natural rate of death, Table 1), and these were not included in the outcome measurements.

**Table 1:** Input data, parameters, and references. [2, 15–26]

Data	Input data	Parameter	Estimate	References
Baseline prevalence of CHC infection	Prevalence of chronic HCV infection in the general population classified by geographic area was 1.4–4.2% from 1994 to 2005, 2.15% in 2004, 2.2% from 2005 to 2008, and 0.97% based on a national survey in 2014			Sunanchaikarn S, et al. 2007; Jatapai A, et al. 2010; Quesada P, et al. 2015; Dynamics of the Thai population data from the Institute for Population and Social Research, Mahidol University.
Recruitment rate of susceptible populations	Total population at the beginning (year 1999)	P_0_	61,623,143	Institute for Population and Social Research, Mahidol University
	Population growth rate (logistic growth curve)	**r**	0.16	Estimated by model
	Influx rate of the population to become susceptible per year	**Flowin**	1.1484 × 10^−8^	Estimated by model
	Maximum population (carrying capacity)	**K**	66,785,001	Institute for Population and Social Research, Mahidol University
Transmission coefficient	Transmission coefficient	**β**	0.327	Hagan H, et al. 2004; Roy E, et al. 2009; Durier N, et. al. 2012; Imran M, et al. 2014.
	Time between exposure onset and chronic infection	**1/ λ**	3.3 years	Hagan H, et al. 2004; Roy E, et al. 2009.
Force of infection	Rate at which high-risk individuals acquire infection (92.5%)	**λ**	Depends on β and the number of population HCV infections during that period, i.e., λ = β × (I / N)	Hansurabhanon T, et al. 2002.
Genotype distribution of HCV in Thailand	Genotype 3: 50.7%; Genotype 1: 30.7%; Genotype 6: 18.6%			Akkarathamrongsin S, et al. 2014
Progression of fibrosis	Fibrosis stage F0 to F1	**f0f1**	0.117	Thein HH, et al. 2008
	Fibrosis stage F1 to F2	**f1f2**	0.085	Thein HH, et al. 2008
	Fibrosis stage F2 to F3	**f2f3**	0.12	Thein HH, et al. 2008
	Fibrosis stage F3 to Cirrhosis Child—Pugh class A (C1)	**f3cA**	0.116	Thein HH, et al. 2008
Progression of cirrhosis	Cirrhosis Child—Pugh class A to B	**cAcB**	0.044	D’Amico G, et al. 2006
	Cirrhosis Child—Pugh class B to C	**cBcC**	0.076	D’Amico G, et al. 2006
Incidence of developing HCC	Cirrhosis stage C1 to HCC_BCLC_A	**c1bA**	0.0068	Benvegnu, L, et al. 2001; Somboon K, et al. 2014.
	Cirrhosis stage C2 to HCC_BCLC_A	**c2bA**	0.0068	Benvegnu, L, et al. 2001; Somboon K, et al. 2014.
	Cirrhosis stage C1 to HCC_BCLC_B	**c1bB**	0.0099	Benvegnu, L, et al. 2001; Somboon K, et al. 2014.
	Cirrhosis stage C2 to HCC_BCLC_B	**c2bB**	0.0099	Benvegnu, L, et al. 2001; Somboon K, et al. 2014.
	Cirrhosis stage C1 to HCC_BCLC_C	**c1bC**	0.0029	Benvegnu, L, et al. 2001; Somboon K, et al. 2014.
	Cirrhosis stage C2 to HCC_BCLC_C	**c2bC**	0.0029	Benvegnu, L, et al. 2001; Somboon K, et al. 2014.
	Cirrhosis stage C1 to HCC_BCLC_D	**c1bD**	0.0068	Benvegnu, L, et al. 2001; Somboon K, et al. 2014.
	Cirrhosis stage C2 to HCC_BCLC_D	**c2bD**	0.0068	Benvegnu, L, et al. 2001; Somboon K, et al. 2014.
	Cirrhosis stage C3 to HCC_BCLC_D	**c3bD**	0.0664	Benvegnu, L, et al. 2001
	Cirrhosis stage C4 to HCC_BCLC_D	**c4bD**	0.0664	Benvegnu, L, et al. 2001
Treatment efficacy	Standard treatment response based on genotype (50–80% based on genotype)	**std_cure**	0.72 (weighted average)	Rosen HR. 2011
	Novel treatment, DAAs based on genotype (96–99% based on genotype)	**new_cure**	0.985 (weighted average)	The AASLD/IDSA/IAS—USA Hepatitis C Guidance 2015
Death rate from cirrhosis and HCC	Death rate for Cirrhosis Child—Pugh class A	**deathcA**	0.01	D’Amico G, et al. 2006
	Death rate for Cirrhosis Child—Pugh class B	**deathcB**	0.2	D’Amico G, et al. 2006
	Death rate for Cirrhosis Child–Pugh class C	**deathcC**	0.57	D’Amico G, et al. 2006
	Death rate for HCC_BCLC_A	**deathbA**	1/(36/12)	EASL-EORTC. 2012
	Death rate for HCC_BCLC_B	**deathbB**	1/(16/12)	EASL-EORTC. 2012
	Death rate for HCC_BCLC_C	**deathbC**	1/(6/12)	EASL-EORTC. 2012
	Death rate for HCC_BCLC_D	**deathbD**	1/(3/12)	EASL-EORTC. 2012
Natural rate of death	Unrelated to cirrhosis and hepatitis C	**natdeath**	0.0424	Estimated by model
Transplantation	Transplantation rate in cirrhosis stage C4	**tranc4**	0.00015	36 cases per year based on data from the organ donation unit, Thai Red Cross
	Transplantation rate in HCC_BCLC_A	**tranbA**	0.00015	
	Transplantation rate in HCC_BCLC_B	**tranbB**	0.00015	
Costs of treatments	Current cost of standard treatment: current price approximately US $100 per week			Data from the general government hospital drug price list in Thailand
	Cost of novel treatment: currently being established			
Treatment coverage	Estimated nationwide HCV treatment was 1000 treatments per year in 2005 and 3000 treatments per year in 2015; PEGylated interferon-based regimen covers persons at fibrosis stages F0–F3 and cirrhosis stage C1)	**std_trF0, std_trF1, std_trF2, std_trF3, std_trC1**	Varied with treatment allocation.	Data were estimated based on data from the Thailand National Health Security Office
			Prioritized treatment scenario: 5%, 5%, 30%, 30%, and 30% of all available treatments for **std_trF0**–**std_trC1**, respectively.	
			Generalized treatment scenario: proportionally distributed to all treatable stages.	
	Direct-acting antiviral agent regimen covers persons at fibrosis stages F0–F3 and cirrhosis stages C1–C4	**new_trF0, new_trF1, new_trF2, new_trF3, new_trC1, new_trC2, new_trC3, new_trC4**	Varied with treatment allocation.	
			Prioritize: 5%, 5%, 15%, 15%, 15%, 15%, 15%, and 15% of all available treatments for **new_trF0**–**new_trC4**, respectively.	
			Generalized treatment scenario: proportionally distributed to all treatable stages	
Initial status of model	Initial proportion of the susceptible population (at risk population)	**init(S)**	0.0316	Estimated by model
	To be used for initializing the model	**init(F0)**	0.2825	Avihingsanon A, et al. 2014
	Proportion of susceptible persons at fibrosis stage F0			
	Proportion of susceptible persons at fibrosis stage F1	**init(F1)**	0.2825	Avihingsanon A, et al. 2014
	Proportion of susceptible persons at fibrosis stage F2	**init(F2)**	0.184	Avihingsanon A, et al. 2014
	Proportion of susceptible persons at fibrosis stage F3	**init(F3)**	0.124	Avihingsanon A, et al. 2014
	Proportion of susceptible persons at Cirrhosis Child—Pugh class A (C1+C2)	**init(CirA)**	0.03175+0.03175	Avihingsanon A, et al. 2014
	Proportion of susceptible persons at Cirrhosis Child—Pugh class B (C3)	**init(CirB)**	0.03175	Avihingsanon A, et al. 2014
	Proportion of susceptible persons at Cirrhosis Child—Pugh class C (C4)	**init(CirC)**	0.03175	Avihingsanon A, et al. 2014
	Initial number of HCC_BCLC_A	**init(HCC_A)**	0	
	Initial number of HCC_BCLC_B	**init(HCC_B)**	0	
	Initial number of HCC_BCLC_C	**init(HCC_C)**	0	
	Initial number of HCC_BCLC_D	**init(HCC_D)**	0	
	Initial number of deaths	**init(death)**	0	
	Initial number of liver transplantations	**init(tranLiv)**	0	
	Initial number of HCC_BCLC cases	**total_HCC**	0	

We compared and evaluated the current standard treatment available in the Thai public health care system (i.e., PEGylated interferon and ribavirin) against new treatments using DAAs (i.e., sofosbuvir-based treatments) in various treatment scenarios. A mathematical model of CHC transmission dynamics was constructed to examine the effectiveness of different treatment policies over the next 20 years.

### Prevalence of CHC infection and susceptible populations

The prevalence of CHC infection in the general population was 1.4%–4.2% in 1994–2005, 2.15% in 2004, 2.2% in 2005–2008, and 0.97% in 2014, according to the 2014 national survey [12, 27, 28]. Susceptible populations were persons with unsafe injection practices, inadequate sterilization of medical equipment in health care settings, and unscreened blood products. The risk of HCV transmission has decreased because of nationwide HCV screening of blood transfusions since 1992 and the discovery of interferon as a treatment for HCV [13, 29]. A public health campaign and improvement in the public health care system has decreased the prevalence of HCV in intravenous drug users who shared needles [13]. The estimated current susceptible population is 1.5% (0.9–1 million cases) based on the dynamic change in the total population at baseline year 2015, the decreasing trend in HCV infections, and conservative estimation of all model parameters. CHC’s transmission dynamics were based on the model of a population with optimal control [15].

### HCV genotypic distribution and degree of liver fibrosis

Most HCV genotypes in Thailand are genotypes 3 (50.7%), 1 (30.7%), and 6 (18.6%) [16]. Weighted average treatment efficacy was included in the model according to genotype for both the PEGylated interferon-based regimen and new antiviral agents (Table 1). Hepatic fibrosis was rated by METAVIR scores [30]. The distribution of liver fibrosis was estimated to be mild (F0–F1), moderate (F2), and severe (F3) in 56.5%, 18.4%, and12.4% of patients, respectively. Cirrhosis was present in 12.7% of the population according to histological data from liver biopsies from 331 Thai patients with CHC [17].

### Natural course of CHC

Of all individuals with acute HCV infection, 18%–36% will spontaneously clear the infection [31]. The model assumes that approximately 70% of individuals who are infected will develop CHC after the start of infection and that for those individuals, no spontaneous clearance occurs after the development of CHC. The fibrosis stage of progression gradually develops stage-by-stage in those with chronic infection [18], and then cirrhosis progresses to a decompensated cirrhosis stage over time. A previous study indicated that the risk of development of HCC was 2.6% and 6.6% yearly for compensated and decompensated cirrhosis, respectively [19]. The distribution of the first diagnosis in the Barcelona Clinic Liver Cancer (BCLC) stage of HCC was estimated to be stage A–D in 25.6%, 37.6%, 11.2%, and 25.6% of patients, respectively [26]. This finding was in the public health context of Thailand, with large numbers of people unaware of their HCV infection and CHC patients not under routine surveillance. Despite treatments, median survival after diagnosis of HCC was estimated to be 36, 16, 6, and 3 months in HCC BCLC Stages A–D, respectively [20].

### Coverage of treatment

The current number of CHC treatment cycles with a PEGylated interferon-based regimen was estimated at 1,000 and 3,000 treatments annually in 2004 and 2014, respectively. A linear increase in treatment was estimated using data from the National Health Security Office of Thailand. This was a conservative coverage scenario. We also constructed an extensive coverage scenario, which was based on reducing the cost of current standard treatment (from approximately 4,500 USD to approximately 900 USD per course of treatment). This scenario might increase treatment accessibility five-fold. However, the cost of treatment with new DAAs still needs to be established. The sensitivity analyses assuming different treatment coverage levels were analyzed according to the conservative coverage scenario, the extensive coverage scenario (5-fold increase), and beyond (10-fold increase).

### Treatment allocation

The current public health strategy of treatment allocation in Thailand, based on the National Health Security Office of Thailand, is to prioritize treatment for advanced fibrosis and compensated cirrhosis groups. We constructed a prioritized treatment scenario based on the current strategy, which allocates more treatment for the advanced stages of disease. We also constructed a generalized treatment scenario, which equally distributes treatment to all HCV patients at all stages of the disease. Data from the Thai Red Cross Organ Donation Center shows that fewer than 100 liver transplants take place in Thailand annually, with only 35% of patients on the waiting list receiving a transplant [32]. This is due to the lack of donated livers. As the number of liver transplants is low it may not affect the overall nationwide burden of CHC infection.

### Risk of development of HCC

The risk of developing HCC is increased after cirrhosis in patients with CHC. Advanced stages of cirrhosis are also associated with an increased chance of developing HCC [19]. Even in cirrhosis patients who achieve a sustained virological response against HCV, the risk of developing HCC is still persistent according to the stage of cirrhosis [33]. We constructed a model and analyzed scenarios with a persistent risk of development of HCC in patients with cirrhosis despite a sustained virological response from treatment.

### Development of a dynamic mathematical model of CHC

A compartmental model with susceptibility, 12 disease states (four fibrosis stages [F0–F3], four cirrhosis stages [C1–C4], and four HCC stages [BCLC A–D]), as well as death was developed to describe the transmission dynamics of HCV (Fig 1). Population flow between compartments is represented by a set of ordinary differential equations derived from data of population dynamics, disease transmission, disease progression, and treatment effects (Table 2).

**Table 2:** Core model equations* derived from data of population dynamics, disease transmission, disease progression, and treatment effects.

$pop=\frac{KP_{0}exp^{rt}}{K+P_{0}\left(exp^{rt}-1\right)}$
*S′ = flowin* × *pop − λS − natdeath* × *S*
*F*0*′* = −*f0f1 × F0* + *λS* − *std_trF*0 × *std_cure* − *new_trF*0 × *new_cure* − *natdeath* × *F*0
*F*1*′* = *f0f1* × *F0 − f1f2* × *F*1 − *std_trF*1 × *std_cure* − *new_trF*1 × *new_cure* − *natdeath* × *F*1
*F*2*′* = *f1f2* × *F1 − f2f3* × *F*2 − *std_trF*2 × *std_cure* − *new_trF*2 × *new_cure* − *natdeath* × *F*2
*F3′* = *f2f3* × *F2 − f3c1* × *F3* − *std_trF*3 × *std_cure*–*new_trF*3 × *new_cure* − *natdeath* × *F*3
*C*1*′* = *f*3*c*1 × *F*3 − *deathc*1 × *C*1 − *c*1*c*2 × *C*1 –*std_trC*1 × *std_cure* – *new_trC*1 × *new_cure* − (*c*1*bA* + *c*1*bB* + *c*1*bC* + *c*1*bD*) × *C*1 − *natdeath × C1*
*C*2*′* = *c*1*c*2 × *C*1 − *deathc*2 × *C*2 − *c*2*c*3 × *C*2 − *new*_*trC*2 × *new*_*cure* − (*c*2*bA* + *c2bB* + *c*2*bC* + *c2bD*) × *C2* − *natdeath × C2*
*C*3*′* = *c*2*c*3 × *C*2 − *deathc*3 × *C*3 − *c*3*c*4 × *C*3 –*new_trC*3 × *new_cure* − *c*3*bD × C*3 − *natdeath* × *C*3
*C*4*′* = *c*3*c*4 × *C*3 − *new*_*tr*C4 × *new*_*cure* − *deathc*4 × *C*4 − *c*4*bD × C*4 − *natdeath × C4*
*HCC*_*A′* = *c*1*bA* × *C*1 + *c*2*bA* × *C*2 − *deathbA* × *HCC*_*A* − *tranbA* × *HCC*_*A* − *natdeath* × *HCC*_*A*
*HCC*_*B′* = *c*1*bB* × *C*1 + *c*2*bB* × *C*2 − *deathbB* × *HCC*_*B* − *tranbB* × *HCC*_*B* − *natdeath* × *HCC*_*B*
*HCC*_*C′* = *c*1*bC* × *C*1 + *c*2*bC* × *C*2 − *deathbC* × *HCC*_*C* − *natdeath* × *HCC*_*C*
$HCC_{D}^{\prime }=c1bD\times C1+c2bD\times C2-deathbD\times HCC_{D}+c3bD\times C3+c4bD\times C4-natdeath\times HCC\_D$
*death′* = *deathc*1 × *C*1 + *deathc*2 × *C*2 + *deathc*3 × *C*3 + *deathc*4 × *C*4 + *deathbA* × *HCC*_*A* + *deathbB × HCC_B* + *deathbC* × *HCC*_*C* + *deathbD* × *HCC*_*D*

*All parameters in equations are defined and described in Table 1.

Most parameters of the model were fixed if the values were estimated by previous studies. The parameters that were estimated by fitting the model to HCV prevalence data were the population growth rate (r), the population’s natural death rate (natdeath), the initial proportion of the population at risk of HCV (S0), and the rate of influx of new susceptibility (flowin). For model calibration, the predicted prevalence was fit to the observed 1994–2014 data using the Berkeley Madonna version 8.3.18 (Berkeley, CA) curve fitting feature. The model with the estimated parameters that yielded the minimum sum of squared residuals or deviation between the model’s predicted prevalence and the actual data was then designated as the baseline model. This model was used to examine different treatments and different treatment allocations.

Two different treatments (i.e., the current treatment [PEGylated interferon and ribavirin] and new treatments using DAAs [sofosbuvir-based treatments]) varied in their efficacy and stage coverage. Treatment responses of PEGylated interferon and DAA regimens were based on the efficacy of each genotype. Weighted average efficacy was used for the dynamic mathematical model (72% and 98.5% in standard and novel treatments, respectively). The PEGylated interferon-based regimen covered persons at fibrosis stages F0–F3 and cirrhosis stage C1. DAA agent regimens covered persons at fibrosis stages F0–F3 and cirrhosis stages C1–C4 (Table 1). These effects of treatment were incorporated into the model, and the effects in terms of HCV prevalence, total deaths, number of deaths related to cirrhosis, and number of HCC-related deaths were compared. The model assumed a persistent risk of development of HCC in patients with cirrhosis despite a sustained virological response from treatment. The numbers of deployed new treatments, classified by coverage type and the proportion of treatment allocation in the model, are shown in Table 3. The model was constructed and run in Berkeley Madonna. Data analysis and graphics were performed in R version 2.14.2 (available at http://www.R-project.org/).

**Table 3:** Number of deployed new treatments classified by coverage type and proportion of treatment allocation.

Treatment scenarios	Year				
	2015	2020	2025	2030	2035
**Number of treatments in conservative coverage (treatments/year)**	3,000	4,000	5,000	6,000	7,000
**Number of treatments in extensive coverage (treatments/year)**	3,000	8,000	13,000	18,000	23,000
**Treatment distribution in prioritized allocation**	90% of all available treatments were allocated to significant fibrosis (F2–F3) and cirrhosis stage				
**Treatment distribution in generalized allocation**	Proportional distribution of treatment at all stages of the disease based on the number of cases in each stage				

### Sensitivity analysis

To minimize the bias associated with including a relatively large number, in comparison to distribution in the general population, of advanced-stage patients’ data from hospital-based studies, the distribution of cases by fibrosis stage was varied from the initial scenario to increase the prevalence of early fibrotic stages (F0–F1) by approximately 40%. The level of treatment coverage was varied from the conservative coverage scenario to the extensive coverage scenario (5-fold increase) and beyond (10-fold increase) according to prioritized allocation.

### Ethical considerations

The protocol of the study was approved of exemption by the Institutional Review Board of the Faculty of Tropical Medicine, Mahidol University, Thailand (MUTM-EXMPT 2015–002).

## Results

**Fig 2:**
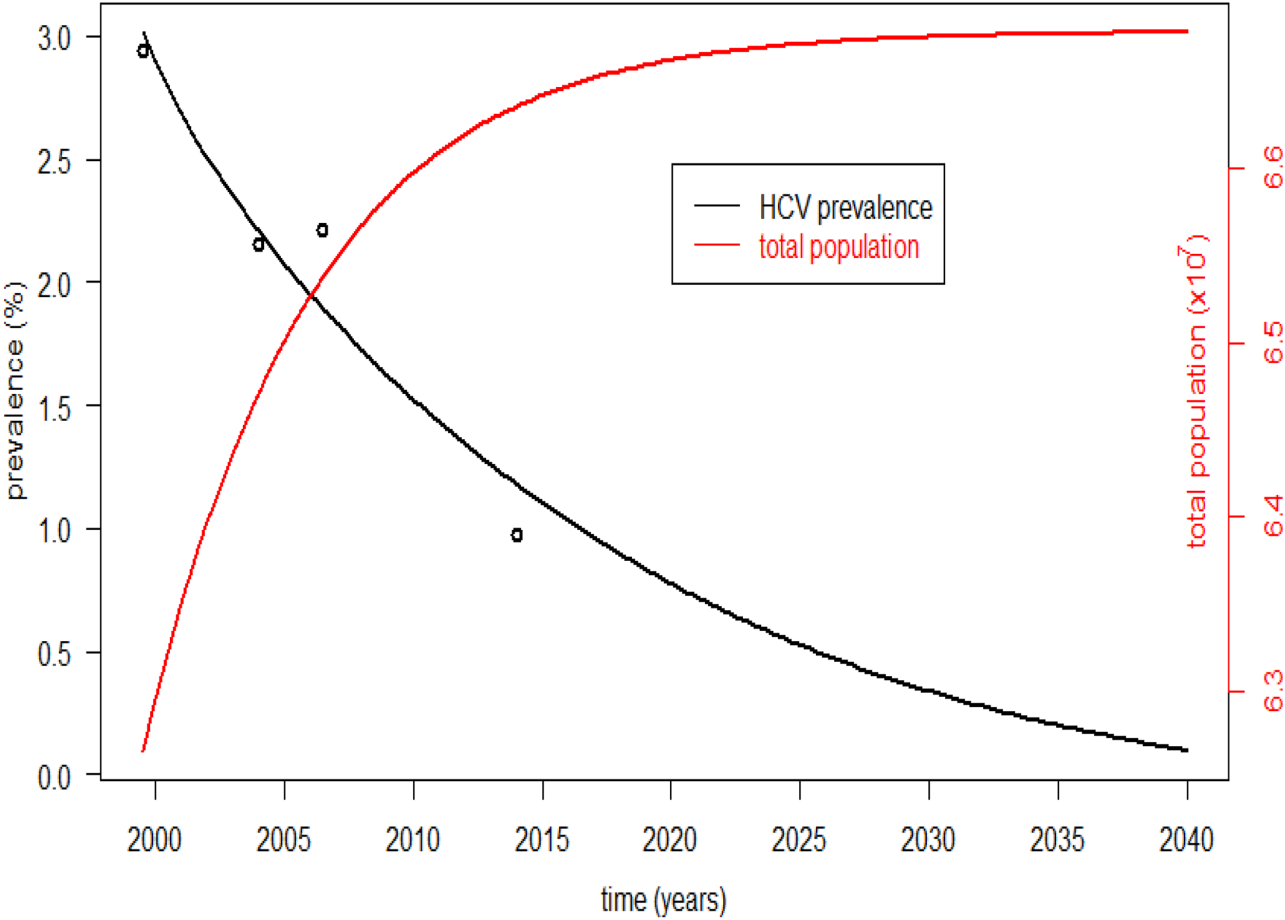
Estimated prevalence of CHC patients. Estimated prevalence of patients with CHC based on previous data and transmission and progression of CHC using current standard treatments in Thailand. The model was created by fitting reported 1994–2014 CHC prevalence data. The reported prevalence of chronic HCV infection in the general population was 1.4%–4.2% in 1994–2005, 2.15% in 2004, 2.2% in 2005–2008, and 0.97% in 2014. Circles represent the observed data, and the black line represents prediction of the model. The right Y-axis represents the estimated population of Thailand derived by the model.

Over the next 20 years, the prevalence of CHC in Thailand was estimated to decrease from 726,000 (1.09%) cases in 2015 to 134,432 (0.196%) cases in 2035 using current standard treatments in the current access situation. The estimated dynamic change in the total population in Thailand will increase at a slower rate after 2015. These data were fit with the changing trend in HCV seroprevalence from 1994–2014 published in a previous report (Fig 2). The best fit was obtained when the deviation between the model’s prediction and observed data (i.e., the root mean square of the differences) was minimized (0.194). Therefore, some model parameters were at their best estimates (Table 1).

**Fig 3:**
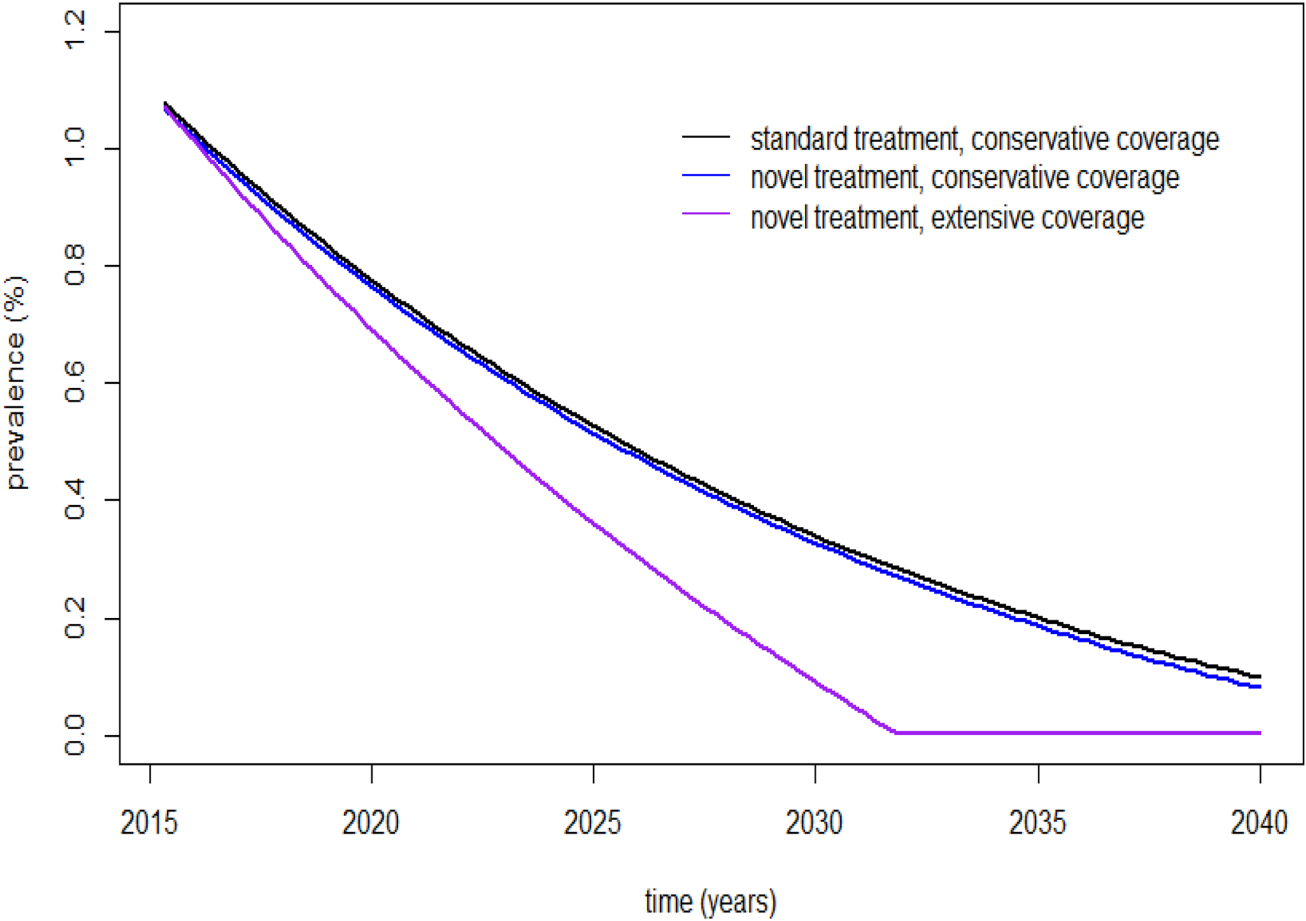
Estimated prevalence of CHC in Thailand over the next 20 years. Estimated prevalence of CHC in Thailand over the next 20 years using current standard treatments and novel antiviral treatments in the conservative coverage scenario and novel antiviral treatments in the extensive coverage scenario.

After DAA treatments were implemented, the prevalence of CHC in Thailand was estimated to decrease to 131,688 (0.192%) cases with the conservative treatment coverage assumption in the next 20 years. Furthermore, the prevalence of CHC in Thailand was estimated to be near zero at that time in the extensive coverage scenario using novel antiviral treatments (Fig 3).

**Fig 4:**
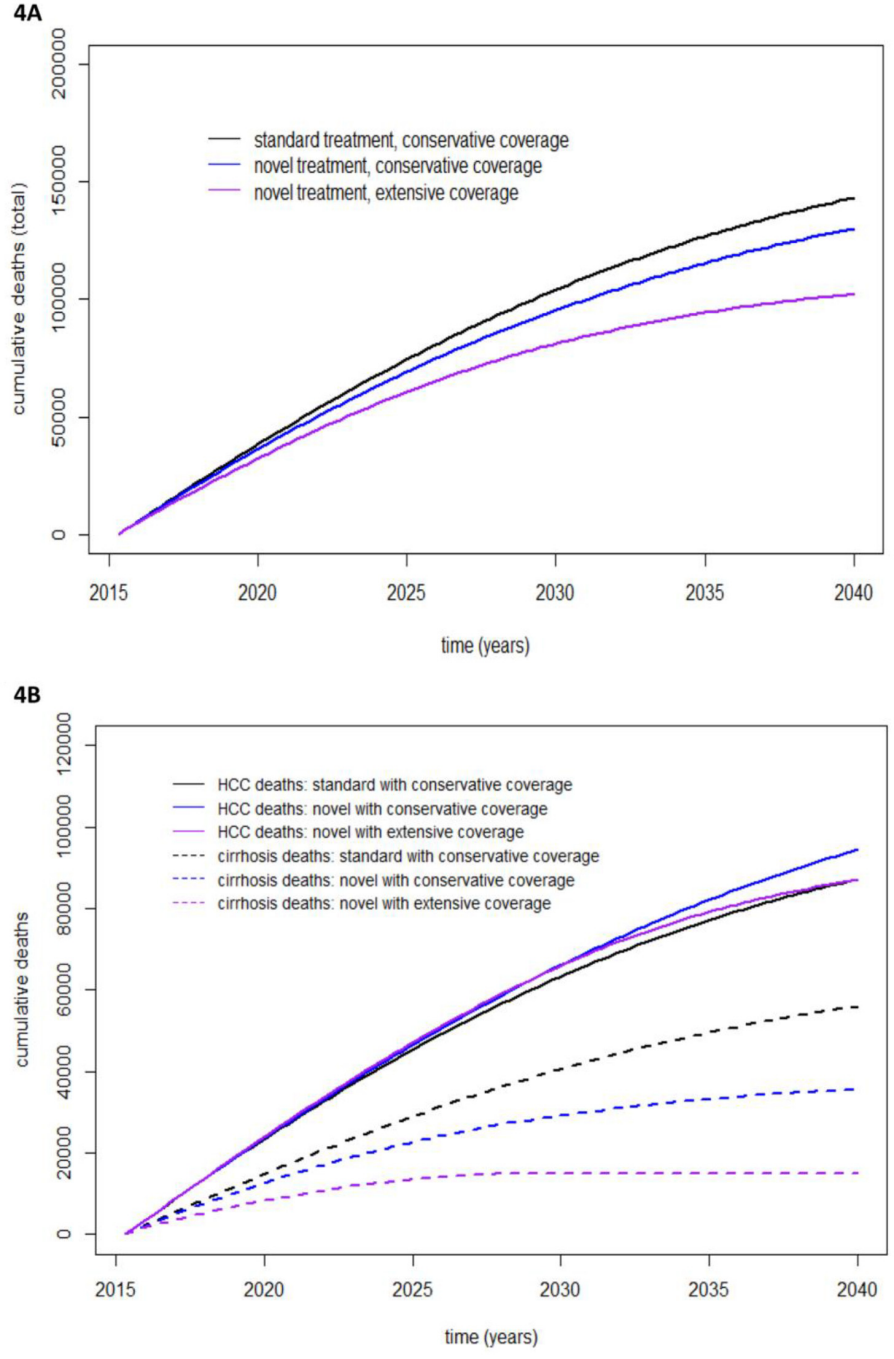
Estimated cumulative death related to HCV. Estimated cumulative total death related to HCV (A) and cumulative death from liver decompensation and HCC related to HCV (B) based on current and extensive treatment coverage over the next 20 years.

Cumulative deaths related to CHC were estimated to decrease 129,685–118,156 (8.9% reduction) after antiviral treatments were implemented with the conservative treatment coverage scenario over the next 20 years. The extensive treatment coverage scenario was estimated to decrease cumulative CHC-related deaths 129,685–96,678 (25.5% reduction). Furthermore, 21,478 lives were estimated to be saved with the extensive treatment coverage scenario compared with the conservative treatment coverage scenario (Fig 4A). Cumulative death related to HCV from decompensated cirrhosis was estimated to decrease 50,751–34,148 (32.7% reduction) cases after the implementation of novel antiviral treatments in the conservative coverage scenario. However, cumulative death related to HCC was predicted to increase slightly, 78,934–84,008 (6.4% increase). The burden of cumulative death related to HCC will increase after 2030 because of the large cumulative number of cases cured of HCV in the cirrhotic stage with a persistent risk of HCC development. The extensive coverage scenario brought about a rapid decrease in cumulative deaths from decompensated cirrhosis (50,751–15,812; 68.8% reduction), but the cumulative number of HCC-related deaths was predicted to increase slightly, 78,934–80,866 (2.4% increase). Death related to decompensated cirrhosis was predicted to stop only in the extensive coverage scenario in 2031 (Fig 4B).

**Fig 5:**
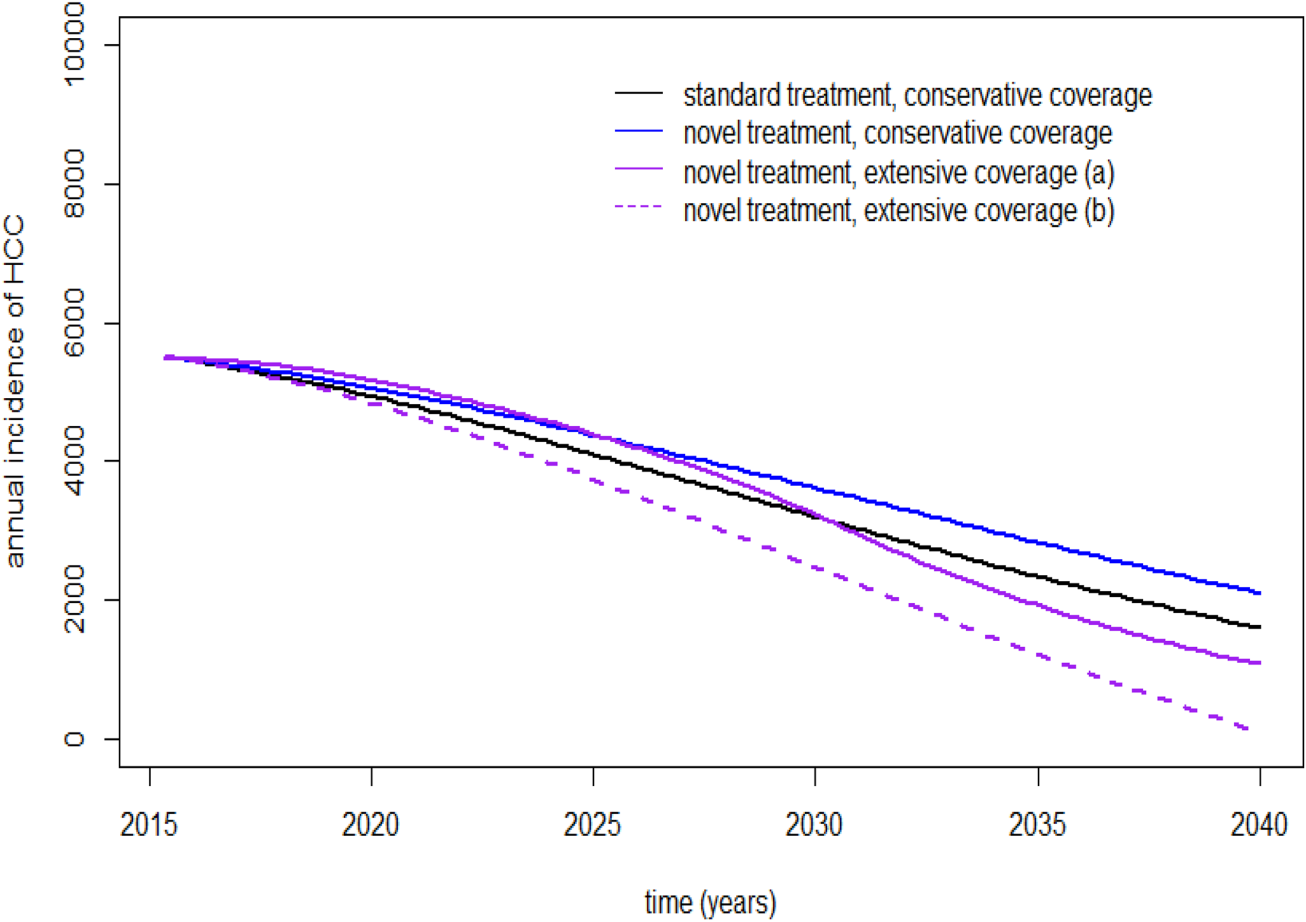
Estimated annual incidence of HCC related to HCV. Estimated annual incidence of HCV-related HCC based on current treatment coverage using standard and novel antiviral treatments, estimating current and extensive treatment coverage over the next 20 years. a: prioritized treatment scenario, b: generalized treatment scenario.

In 2015, the annual incidence of HCC-associated HCV was estimated to be 5,524 cases annually. Over the next 20 years, the yearly incidence of HCC-associated HCV was estimated to decrease to 2,305 annual cases in the current coverage situation. After implementing novel antiviral treatments, this will result in an increase in the yearly incidence of HCC-associated HCV: 2,305–2,798 cases yearly (21.4% increase) in the conservative coverage scenario. However, the annual incidence of HCC-associated HCV was estimated to decrease to 1,877 cases annually (18.6% reduction) in the extensive treatment coverage scenario. The annual incidence of HCC will considerably decrease in the extensive treatment coverage scenario after 2032 because of a decreased prevalence of the cirrhotic stage after that time (Fig 5).

Various effects on the HCV-associated burden were observed upon replacing the prioritized treatment scenario (current standard public health policy in Thailand) with the generalized treatment scenario (treatment proportionally allocated to all disease stages). After implementing the generalized treatment scenario, the predicted number of cumulative deaths from decompensated cirrhosis increased 34,148–48,202 (41.2% increase) in the conservative treatment coverage scenario and 15,812–28,609 (80.9% increase) in the extensive treatment coverage scenario over the next 20 years. In the conservative treatment coverage scenario, the generalized treatment scenario did not appear to decrease overall death from decompensated cirrhosis and CHC-related HCC (118,156 vs. 126,777, respectively). In the extensive treatment coverage scenario, the generalized treatment scenario considerably reduced HCC-related death 80,866–71,696 (11.3% reduction) but increased overall death 96,678–100,304 (3.8% increase). However, the annual incidence of HCC-associated HCV was predicted to decrease further, 1,877–1,168 cases yearly (37.7% reduction). The highest absolute number of lives estimated to be saved occurred in the prioritized treatment scenario with extensive treatment coverage (Table 4).

**Table 4:** Estimated HCV-associated burden over the next 20 years classified by treatment regimen, treatment coverage, and allocation strategy.

	CHC prevalence (%)	Annual HCC incidence	Death from liver cirrhosis	HCC-related death	Total death
**Current standard treatment, conservative coverage, prioritized allocation**	0.196 (baseline)	2,305 (baseline)	50,751 (baseline)	78,934 (baseline)	129,685 (baseline)
**Novel antiviral treatment, conservative coverage, prioritized allocation**	0.192 (−2.0%)	2,798 (+21.4%)	34,148 (−32.7%)	84,008 (+6.4%)	118,156 (−8.9%)
**Novel antiviral treatment, extensive coverage, prioritized allocation**	0.002 (−98.9%)	1,877 (−18.6%)	15,812 (−68.8%)	80,866 (+2.4%)	96,678 (−25.5%)
**Current standard treatment, conservative coverage, generalized allocation**	0.195 (−0.5%)	2,346 (+1.7%)	50,934 (+0.4%)	79,221 (+0.4%)	130,156 (+0.4%)
**Novel antiviral treatment, conservative coverage, generalized allocation**	0.176 (−10.2%)	2,242 (−2.7%)	48,202 (−5.0%)	78,575 (−0.5%)	126,777 (−2.2%)
**Novel antiviral treatment, extensive coverage, generalized allocation**	<0.001 (−99.6%)	1,168 (−49.3%)	28,609 (−43.6%)	71,696 (−9.2%)	100,304 (−22.7%)

According to the extensive coverage scenario using novel antiviral treatments, we estimated a diminished incidence of HCC-related CHC after 2040 in the generalized treatment scenario, with an annual incidence of less than 100 cases in 2040. This was predicted to continue decreasing thereafter in the prioritized treatment scenario (Fig 5).

**Fig 6:**
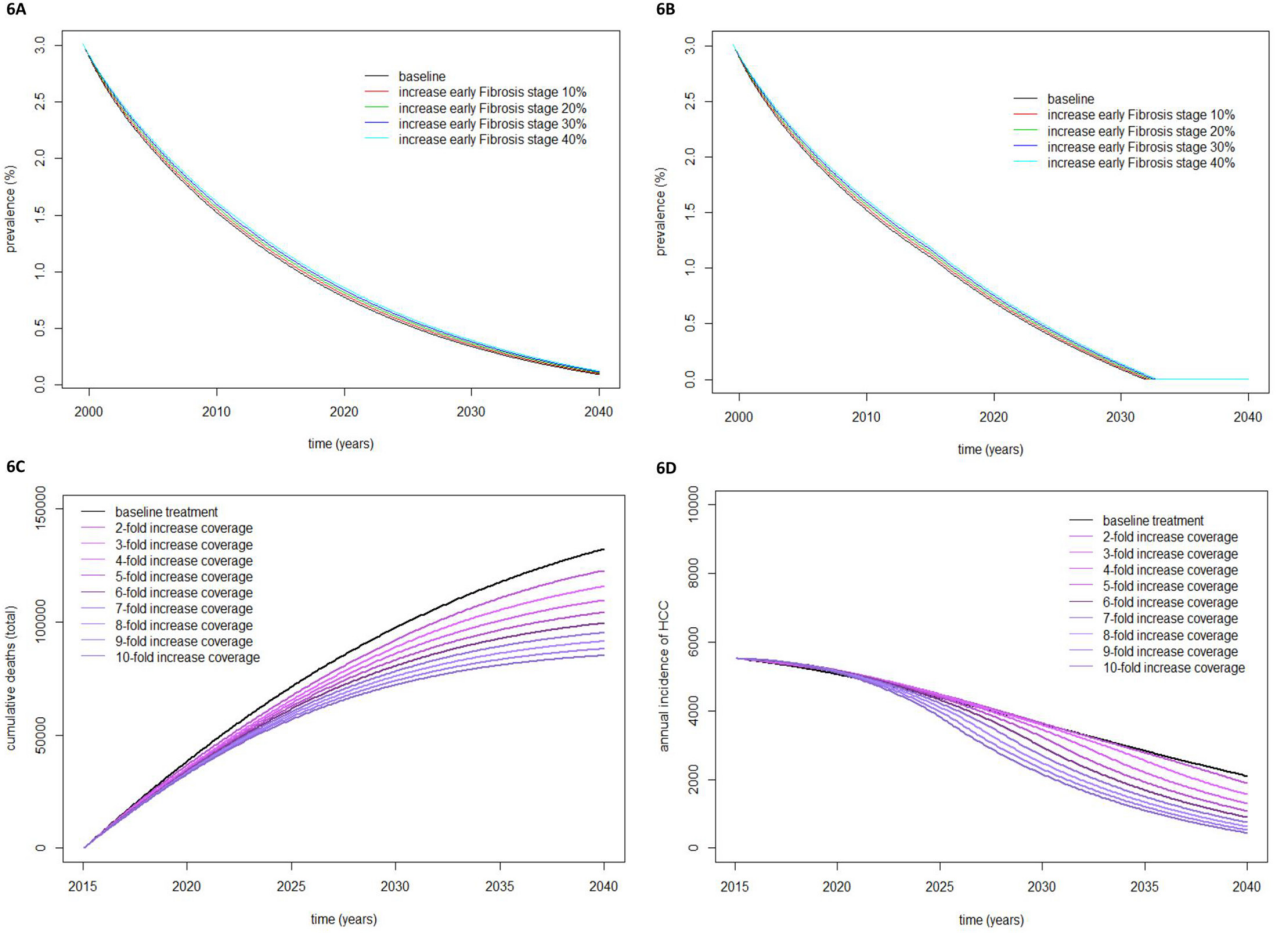
Sensitivity analysis. Estimated CHC prevalence with different case distributions by fibrosis stage in conservative treatment coverage scenario (A), estimated prevalence of CHC with different case distributions by fibrosis stage in extensive treatment coverage scenario (B), estimated cumulative overall CHC-related death with different levels of treatment coverage (C), and estimated annual incidence of HCC-associated HCV with different levels of treatment coverage (D).

Based on sensitivity analysis of fibrosis stage, the predicted prevalence of CHC in 2035 varied 0.195%–0.237% with the novel antiviral treatment in a conservative treatment coverage scenario (Fig 6A). CHC prevalence in 2035 is not predicted to differ between the conservative and extensive treatment coverage scenarios and is estimated at 0.002% (Fig 6B).

Based on sensitivity analysis of treatment coverage, cumulative overall CHC-related death by 2035, varied 117,817–80,971 (31.2% difference; Fig 6C), and the annual incidence of HCC-associated HCV varied 2,798–1,056 cases yearly (Fig 6D).

## Discussion

This study demonstrated the use of a simple model-based approach to study the effects of comparative treatment strategies for CHC, which indicated a decrease in the CHC burden in the next 20 years in Thailand. Treatment policy, including expanding treatment coverage and strategic treatment allocation, had a large effect on the CHC burden. Over the next 20 years, expanding coverage will decrease the estimated prevalence of CHC from 0.19% in the conservative coverage scenario to almost zero in the extensive coverage scenario. New DAAs are beneficial for reducing the death rate related to decompensated cirrhosis. Expanding coverage will decrease HCC’s future incidence and burden.

Because of the persistent risk of development of HCC after a sustained virological response in cirrhosis patients, [33] HCC-related burden will considerably diminish only in the extensive coverage scenario. Morbidity and mortality related to CHC in Thailand were estimated to decrease over the next 20 years. Treatment coverage had a large effect on overall morbidity and mortality in our model, but treatment costs can be a major limitation to expanding treatment coverage.

CHC is still a global public health burden because of a lack of awareness in most of the infected population and a lack of access to treatment in many countries owing to the unavailability of affordable drugs and the cost and complexity of diagnostics and staging methods such as molecular diagnosis and liver fibrosis testing. The need to improve access to care and treatment for chronic HCV infection in resource-limited settings is a global issue. The main priorities for scaling up HCV treatment and care include reducing the cost of treatment, simplifying the package of care, identifying opportunities to shift specific tasks to non-specialist doctors to overcome human resource constraints, and expanding treatment coverage [34].

Strategies for treatment to decrease morbidity from HCV-related liver cirrhosis might differ from those for the prevention of HCC development. After patients develop cirrhosis, the risk of developing HCC might not decrease [33]. We showed that the incidence of HCC development will not decrease with new DAA treatments without the expansion of treatment coverage. Furthermore, the incidence of HCC will considerably decrease further in the next 20 years because of generalized treatment policies.

We estimate that HCV infection and its related disease burden will be diminished within 23 years; however, elimination of HCV may not be achieved where access to treatment is limited. According to the literature, 90% of individuals infected with HCV worldwide reside in resource-limited settings [6]. Of note, even in a developed country such as the USA, less than half of those infected with HCV are aware of their infection. These findings suggest that more intensive efforts are required to identify and test persons at risk of HCV infection [35]. Even as diagnosis rates increase, it is likely there will be undiagnosed cases that remain; these will be difficult and therefore expensive to reach. Developing a strategic program to examine unrecognized HCV and determine those who are at risk of HCV infection, in order to increase the screening and treatment uptake rate, is crucial to the elimination of CHC infection. Our model assumed that the population had full access to treatment and that patients fully complied with treatment, but this may not be possible in most settings.

Emerging viral resistance to DAAs in the future is a limitation that might interfere with this study’s estimated results. A previous report showed possible genetic resistance to new DAAs [36]. Therefore, there is a need to monitor the development of widespread, clinically resistant clones of HCV, which might affect global HCV elimination. Treatment efficacy was assumed to be consistent and not affected by the emergence of antiviral resistance over the studied time frame in our model.

Patients in resource-limited countries, including Thailand, are currently unable to access new treatments. There are often dilemmas about the decision to treat CHC patients with a PEGylated interferon-based regimen or to wait for new drugs, especially in countries where novel treatments are currently unavailable. Apart from those who benefit from interferon-free therapy, the superiority of health benefits is lost when the waiting time for interferon-free therapy is greater than 3 years [37].

Massive screening of blood products, a decreasing population of intravenous drug users, and the availability of HCV treatments might contribute to HCV’s decreasing prevalence. The transmission parameter in this study is based only on data from studies following people who inject drugs as there is limited nosocomial transmission dynamic information available for Thailand. It is possible that this leads to an overestimation of new CHC cases. A recent study estimated a decrease in the prevalence of HCV infection of 0.5%–1.2% in adults during 1990–2005 in Southeast Asia [6].

Despite the high diversity of Southeast Asia’s HCV genotypic distribution [38], this analysis will have enhanced utility soon. After 2015, the upcoming economic integration of the Association of Southeast Asian Nations will allow unrestricted travel among residents of the member states and unprecedented free trade of goods and services, including healthcare. This study may provide justifications for sound public health policy related to HCV treatment in the future, especially in Southeast Asia.

Current treatment policy based on Thailand’s National Reimbursement Policy to treat patients with significant fibrosis (METAVIR fibrosis score ≥2 or comparable noninvasive methods) might not prevent further transmission. This is because the age of transmission is usually in early adulthood, and a long time may pass before patients develop clinically significant fibrosis or cirrhosis. Although new treatments will become available worldwide, campaigns to improve awareness and reduce transmission need to be scaled up to prevent a future rise in mortality [39].

The limitations of the model are caused by deficiencies in the currently available data and some parameters that need to be estimated by the model. Some possible emerging parameters, such as viral resistance to DAAs, were also not included in the model. Some input data are uncertain because of the limits of currently available data, such as distribution of fibrosis stage and treatment coverage after reducing treatment cost. However, we have conducted a sensitivity analysis to explore these parameters’ effects on the outcomes. Based on our sensitivity analysis, more cases at the early fibrosis stage predicted a higher prevalence of CHC due to a decrease in mortality effect.

The prevalence of CHC was estimated to decrease over time. Enhancing treatment coverage will accelerate disease elimination. Treatment before the cirrhosis stage will affect the long-term incidence of CHC-related HCC. Enhancing treatment coverage and generalized treatment policy will also affect future HCC incidence. New antiviral treatments can significantly extend life in advanced disease stages, but only with enhanced treatment coverage. A decrease in future HCC-related burden could be extended further through use of generalized treatment allocation.

Reduction of future HCC incidence and HCV transmission are indirect benefits of expanding CHC coverage and a generalized treatment policy. Emphasis on treatment benefits beyond preventing deaths from decompensated cirrhosis and the effects of public health policies are essential for public health care policy makers, especially in resource-limited countries.

After the novel DAA era, we estimate that a near-zero incidence of death from decompensated cirrhosis will occur in 15 years in Thailand in the ideal treatment coverage and allocation scenario. However, potential barriers to this near-zero incidence include immigration of persons with advanced HCV-related liver disease into the country and the lack of capacity to carry out mass screening across all susceptible groups.
